# The Prevalence of Adherent-Invasive *Escherichia coli* and Its Association With Inflammatory Bowel Diseases: A Systematic Review and Meta-Analysis

**DOI:** 10.3389/fmed.2021.730243

**Published:** 2021-12-03

**Authors:** Razie Kamali Dolatabadi, Awat Feizi, Mehrdad Halaji, Hossein Fazeli, Peyman Adibi

**Affiliations:** ^1^Department of Microbiology, School of Medicine, Isfahan University of Medical Sciences, Isfahan, Iran; ^2^Department of Biostatistics and Epidemiology, School of Health, Isfahan University of Medical Sciences, Isfahan, Iran; ^3^Infectious Diseases and Tropical Medicine Research Center, Babol University of Medical Sciences, Babol, Iran; ^4^Department of Microbiology, School of Medicine, Babol University of Medical Sciences, Babol, Iran; ^5^Gastroenterology and Hepatology Research Center, Isfahan University of Medical Sciences, Isfahan, Iran

**Keywords:** adherent-invasive *Escherichia coli*, inflammatory bowel diseases, meta-analysis, AIEC, phylogroup

## Abstract

Inflammatory bowel diseases (IBD), including Crohn's disease (CD) and ulcerative colitis (UC), are known as chronic gastrointestinal inflammatory disorders. The present systematic review and meta analysis was conducted to estimate the prevalence of adherent-invasive *Escherichia coli* (AIEC) isolates and their phylogenetic grouping among IBD patients compared with the controls. A systematic literature search was conducted among published papers by international authors until April 30, 2020 in Web of Science, Scopus, EMBASE, and PubMed databases. The pooled prevalence of AIEC isolates and their phylogenetic grouping among IBD patients as well as in controls was estimated using fixed or random effects models. Furthermore, for estimating the association of colonization by AIEC with IBD, odds ratio along with 95% confidence interval was reported. A total of 205 articles retrieved by the initial search of databases, 13 case–control studies met the eligibility criteria for inclusion in the meta analysis. There were 465 IBD cases (348 CD and 117 UC) and 307 controls. The pooled prevalence of AIEC isolates were 28% (95% CI: 18–39%), 29% (95% CI: 20–40%), 13% (95% CI: 1–30%), and 9% (95% CI: 3–19%), respectively among IBD, CD, UC, and control group, respectively. Our results revealed that the most frequent AIEC phylogroup in the IBD, CD, and control groups was B_2_. Fixed-effects meta analysis showed that colonization of AIEC is significantly associated with IBD (OR: 2.93; 95% CI: 1.90–4.52; *P* < 0.001) and CD (OR: 3.07; 95% CI: 1.99–4.74; *P* < 0.001), but not with UC (OR: 2.29; 95% CI: 0.81–6.51; *P* = 0.11). In summary, this meta analysis revealed that colonization by AIEC is more frequent in IBD and is associated with IBD (CD and UC). Our results suggested that the affects of IBD in patients colonized with the AIEC pathovar is not random, it is in fact a specific disease-related pathovar.

## Introduction

Inflammatory bowel diseases (IBD), including Crohn's disease (CD) and ulcerative coliti (UC), are chronic incurable gastrointestinal inflammatory disorders with unknown etiology (1). Previously, IBDs were regarded as a disease prevalent in industrialized countries. However, in the 21st century, as the epidemiological trend of this disease changed, IBDs have become a global problem, and new cases in developing countries in Asia, South America, and Africa are on the rise (2).

They are probably the result of improper and continuous initiation of the intestinal mucosal immune system due to the complicated interactions of genetics, microbial, and immunological agents (3).

As shown by previous experimental and clinical research, intestinal bacteria play a role in the development of IBD and the severity of the disease (4). According to the recent molecular studies on patients with IBD, beneficial bacteria, for instance, Firmicutes and Bacteroidetes, have reduced, and pathogenic bacteria, for example, Proteobacteriae, particularly *Escherichia coli*, have increased (5).

Recently, the links between IBD and adherent-invasive *Escherichia coli* (AIEC) strains have been discussed (6–9). It is possible to classify *E. coli* strains into commensal or pathogenic categories based on genetic and phylogenetic characteristics. The definition of the pathogenic potential of *E. coli* is changing to some extent. AIEC was recently recognized as a pathotype of *E. coli* (late 1990s), and the variable range of AIEC was different from the six primary diarrheagenic *E. coli* pathotypes (Enteropathogenic *E. coli* (EPEC), Enteroinvasive *E. coli* (EIEC), Shiga toxin-producing *E. coli* (STEC), diffusely adherent *E. coli* (DAEC), Enterotoxigenic *E. coli* (ETEC), and Enteroaggregative *E. coli* (EAEC) (10). AIEC is well-known for its role in clinical and experimental epidemiological studies in IBD pathogenesis (11–14). These strains have the ability of adherence and invasion to intestinal epithelial cells (IECs) and extensive survival in macrophages by secreting high levels of tumor necrosis factor alpha (TNF-a) (15). Adherence of this pathotype through type 1 pili expression in the bacterial surface and through cell adhesion molecule 6 (CEACAM6) is attributed to the presence of carcinoembryonic antigen in the ileal epithelial cells' apical surface (16, 17). CEACAM6 is an AIEC receptor that has been indicated to have an abnormal expression in the ileal epithelial cells of adult CD patients (18).

Genetically, AIECs are very close to extraintestinal pathogenic *E. coli*, which includes uropathogenic *E. coli* as well as neonatal meningitis-related strains (5, 19). Based on the strong evidence concerning AIEC role in the promotion of gut inflammation and exacerbation of IBD pathology, most of the AIEC isolates belong to the D and B_2_ phylogenetic groups of *E. coli*, as shown by genomic studies. In addition, based on the distribution of AIEC strains from the phylogenetic perspective, the dominant force in the formation of this pathotype is convergent evolution (11, 12). Previous research has shown that AIEC encodes a large subunit of propandiol dehydratase (a fermentation product of 1,2-propandiol fucose) that is elevated in the microbiome of patients with CD and directs intestinal T cell inflammation induced by AIEC (20).

Moreover, Rath et al. reported that the main role of AIEC in Crohn's disease is to impair the mitochondrial function of epithelial cells. Additionally, different types of intestinal inflammation have shown mitochondrial dysfunction, and it has been indicated that mutations in mitochondria modulating genes are susceptible to IBD (21).

To enhance information about the epidemiology of AIEC in IBD, comprehensive study on the prevalence of AIEC in IBD patients worldwide is believed to be of great value. Therefore, the current systematic review and meta analysis aimed to investigate the prevalence of AIEC isolates and their phylogenetic in IBD patients compared with the controls and the association of colonization by AIEC with IBD, which was quantified by estimating pooled odds ratio (OR) along with 95% confidence interval for OR.

## Methods

### Search Strategies

The research design in the present work followed the preferred reporting items for systematic reviews and metaanalyses (PRISMA) procedures (Supplementary Table 1). The Web of Science, Scopus, EMBASE, and PubMed databases were used for a systematic literature review. The papers published by international authors up to end of April 30, 2020 were searched and reviewed. The following terms were searched as keywords of the present research: “AIEC” or “adherent-invasive *Escherichia coli*” AND “IBD” or “inflammatory bowel disease” OR “CD” or “Crohn's disease” OR “UC” or “ulcerative colitis” without restricting the country. We used the papers that reported the AIEC frequency/prevalence or distribution and their phylogenetic classification in patients with IBD and control group for conducting a comprehensive search. The studies in any language from any region were investigated.

### Inclusion and Exclusion Criteria

For determining eligibility of studies for meeting the inclusion criteria, the databases with related key terms were independently screened by two reviewers, and the titles, abstracts, and full texts were reviewed, and any inconsistencies were fixed by consensus. The inclusion criteria included: (1) the case–control research works, cohort, and retrospective studies of patients and control group with diagnosis of IBD, (2) studies reporting the AIEC prevalence in IBD patients using biopsy sample from intestine parts (colon and ileum), invasion assay, bacterial adhesion, bacterial survival, and replication in macrophages approaches for AIEC detection). We excluded clinical trials, meta analysis, review, or systematic article, case studies, editorials, letters to the editors, abstracts of meetings, congress, and non-human studies. Only biopsy samples were analyzed, and AIEC levels among the other intestinal bacteria are not mentioned.

### Quality Assessment and Data Extraction

The quality of studies was evaluated independently by two authors (RD and MH) using the quality assessment tool for case–control studies developed by Joanna Briggs Institute (JBI), and disagreements were resolved by the third author (PA). Item-related title and abstract, introduction, methods, results, discussion, and other information were determined and a score was assigned to each item. Studies with a score greater than or equal to 60% were included.

Finally, detailed information on eligible studies including the first authors name, publication date, place of study, population studied (IBD patient and control group), type of sample (biopsy), sample size in both IBD and control groups, and sample size of AIEC and phylogroups analysis were extracted.

### Statistical Analysis

The pooled prevalence of AIEC isolates and their phylogenetic in IBD patients and controls along with 95% confidence intervals (95% CI) was estimated by applying the “metaprop program” in STATA statistical software. In this meta analysis, confidence interval for proportion was computed by using score method. In all included studies, we evaluated the association of AIEC with IBD, and the prevalence of AIEC was compared between patients and control groups, and for quantifying the association of colonization by AIEC with IBD, the odds ratio (OR) and 95% confidence interval (95% CI) for OR was calculated as the pooled estimate of effect size using the DerSimonian and Laird method (22). Statistical heterogeneity between studies was evaluated using the Cochran Q Chi-squared test and Cochrane-I-square, and values of 25, 50, and 75% for *I*^2^ were considered as low, medium, and high levels of heterogeneity, respectively (23). When *P*-value <0.10 for Cochran Q Chi-squared test and the value of Cochrane-*I*^2^ was more than 50%, the heterogeneity was considered as high and a random effect approach was adopted for estimating the pooled prevalence, OR, and confidence intervals. The funnel plot, Begg's rank correlation test, and Egger's weighted regression tests were performed to evaluate possible publication bias, and any appeared asymmetry in funnel plot or *P* < 0.05 in used tests was considered as indication of statistically significant publication bias (24). Possible sources of heterogeneity were examined using sensitivity analysis and meta regression to evaluate the confounding role of age. Moreover, sensitivity analyses were conducted to determine the extent to which inferences (the estimated pooled prevalence and OR) might be related to a particular study. All statistical analyses were performed, using STATA Version 11 (Stata Corp., College Station, TX, USA). *P*-values <0.05 were considered statistically significant.

## Results

A total of 205 articles were retrieved by the initial search of databases, of which 182 were removed following selection based on titles, abstract, and index review, and 23 studies were selected for full-text analysis. After assessment of the 23 reviewed studies, three studies collected samples from stool specimen (9, 25, 26), one study had a methodology problem (27), one study collected samples from animal sources (28), two studies did not report the prevalence of AIEC isolates (12, 29), results of a study was unclear in terms of the number of patients and biopsy samples (30), one study was performed on standard isolates (6), and one study did not report the results of control group (31).

**Figure 1:**
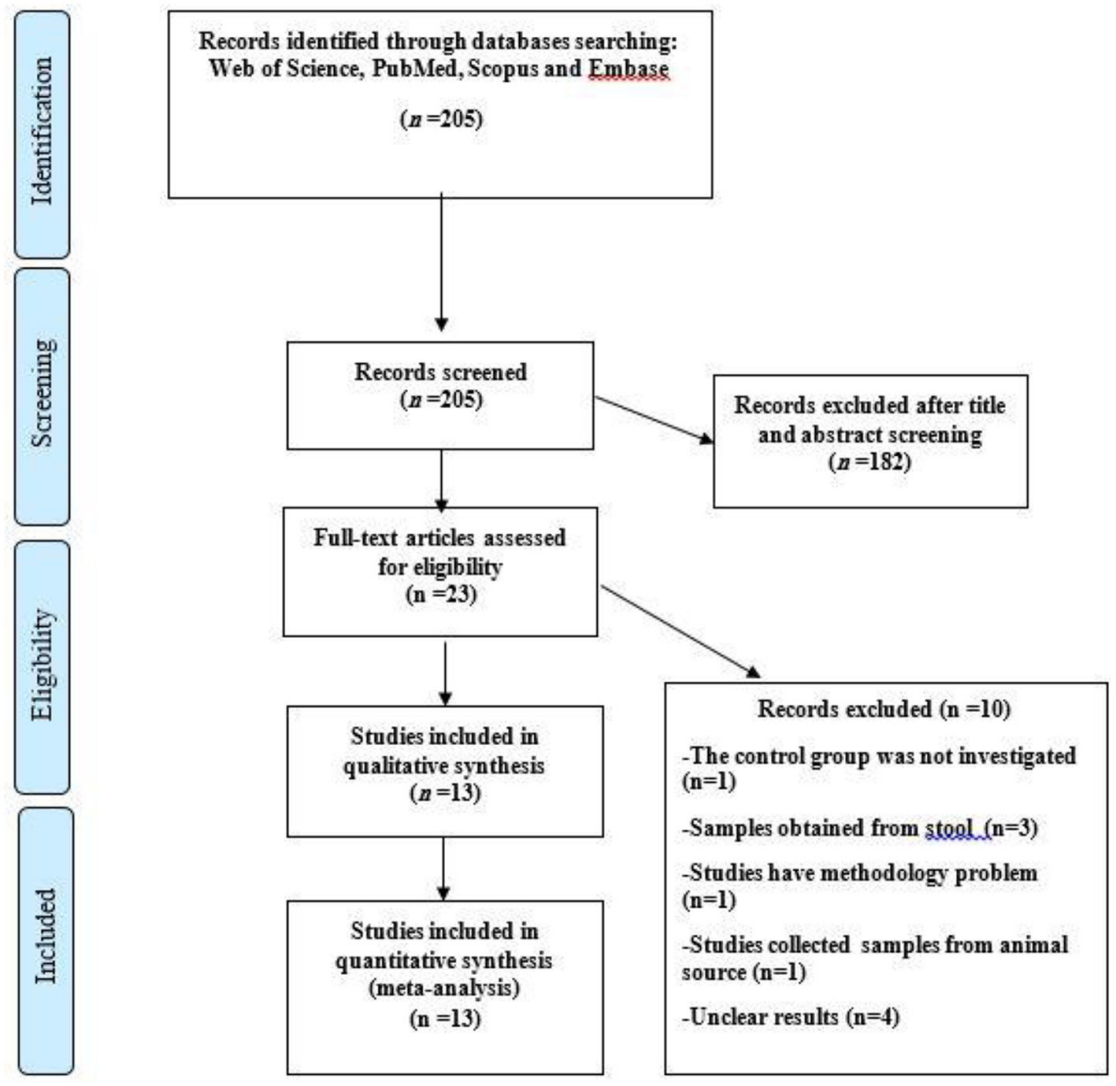
Flow chart of the study selection for inclusion in the systematic review.

Finally, 13 case–control studies met the eligibility criteria for inclusion in the meta analysis. Figure 1 shows a flow diagram illustrating the searching procedure for the selection of eligible studies (16, 32–43). Also, the detailed features of the included articles are accessible in Tables 1, 2. All of the included studies used intestine biopsy samples, bacterial adhesion, invasion assay, bacterial survival, and replication in macrophages methods for the detection of AIEC. All of the articles were case–control studies published between 2004 and 2020.

**Table 1:** The characteristics of studies included in the systematic review.

	**Study**	**Publication year**	**Location**	**Population studied**	**Type of sample**	**IBD**		**CD**		**UC**		**Control**	
						**SS of patients**	**SS of AIEC**	**SS of patients**	**SS of AIEC**	**SS of patient**	**SS of AIEC**	**SS of patient**	**SS of AIEC**
1	Darfeuille-Michaud et al.	2004	France	IBD	B	90	18	90	18	-	-	118	3
2	Baumgart et al.	2007	USA	IBD	B	21	10	21	10	-	-	7	1
													
3	Medina et al.	2009	Spain	IBD	B	20	11	20	11	-	-	28	6
													
4	Raso et al.	2011	Italy	IBD	B	14	4	8	4	6	0	4	0
5	Negroni et al.	2012	Italy	IBD	B	34	2	24	1	10	1	23	0
6	Dogan et al.	2013	New York	IBD	B	32	8	32	8			28	5
7	Elliott et al.	2013	UK	IBD	B	45	2	30	2	15	0	14	0
8	Fuente et al.	2014	Chile	IBD	B	91	8	34	6	57	2	22	0
													
9	O'Brien et al.	2016	Australian	IBD	B	19	5	14	3	5	2	21	5
10	Cespedes et al.	2017	Spain/USA	IBD	B	24	13	24	13			8	0
11	Font et al.	2019	Spain	IBD	B	33	15	33	15	-	-	25	6
12	Lee et al.	2019	Korea	IBD	B	42	14	18	5	24	9	9	2
13	Abdelhalim et al.	2020	Turkey	IBD	B	24	10	24	10			15	7

*B, Biopsy; CD, Crohn's disease; UC, ulcerative colitis; IBD, Inflammatory bowel disease; SS, Sample size*.

**Table 2:** The details of distribution of AIEC based IBD, CD, UC, and Control.

**Study**	**IBD**				**CD**				**UC**				**Control**			
	**SS of patients/AIEC**	**Location of sample AIEC (positive sample)**			**SS of patients/AIEC**	**Location of sample AIEC (positive sample)**			**SS of patients/AIEC**	**Location of sample AIEC (positive sample)**			**SS of patients/AIEC**	**Location of sample AIEC** **(positive sample)**		
Darfeuille-Michaud et al.	90/18	Ileal	Colon		90/18	Ileal	Colon		-	-			118/3	Ileal	Colon	
		63 (17)	27 (1)			63 (17)	27 (1)							16 (1)	102 (2)	
Baumgart et al.	21/10	Ileal			21/10	Ileal			-	-			7/1	Ileal		
		21 (10)				21 (10)								7 (1)		
Medina et al.	20/11	Ileal	Colon	Ileal + colon	20/11	Ileal	Colon	Ileal + colon	-	-			28/6	Ileal	Colon	Ileal + colon
		4 (4)	9 (6)	16 (1)		4/4	9/6	7/1						9 (3)	11 (3)	8 (0)
Raso et al.	14/4	ND			8/4	ND			6/0	ND			4/0	ND		
Negroni et al.	34/2	Ileal			24/1	Ileal			10/1	Colonic			23/0	-		
		34 (2)				24 (1)				10(1)					
Dogan et al.	32/8	Ileal			32/8	Ileal			-	-			28/5	Ieal		
		32 (8)				32 (8)								28 (5)		
Elliott et al.	45/2	ND			30/2	ND			15/0	ND			14/0	ND		
Fuente et al.	91/8	Ileal			34/6	Ileal			57/2	Ileal			22/0	Ileal		
		91 (8)				34 (6)				57 (2)				22 (0)		
O'Brien et al.	19/5	Terminal Ileumm			14/3	Terminal-Ileumm			5/2	Terminal Ileumm			21/5	Terminal ileumm		
		19 (5)				14 (3)				5 (2)				21 (5)		
Cespedes et al.	24/13	ND			24/13	ND			-	-			8/0	ND		
Font et al.	33/15	ND			33/15	ND			-	-			25/6	ND		
Lee et al.	42/14	Ileal	Ileocecal valve	Colon	18/5	Ileal	Ileocecal valve	Colon	24/9	Ileal	Ileocecal valve	Colon	9/2	Ileal	Ileocecal valve	Colon
		5(ND)	10(ND)	27(ND)		5(ND)	7(ND)	6(ND)		0(ND)	3(ND)	21(ND)		0(0)	0(0)	9 (2)
Abdelhalim et al.	24/10	Ileal	Colon	Ileocolonic	24/10	Ileal	Colon		Ileocolonic	-			15/7	Ileal	Colon	Ileocolonic
		4(ND)	12(ND)	8(ND)		4(ND)	12(ND)		8(ND)					ND	ND	ND

*CD, Crohn's disease; UC, ulcerative colitis; IBD, Inflammatory bowel disease; SS, Sample size; ND, No Data*.

Totally, there were 465 IBD cases (348 CD and 117 UC) and 307 controls. Given that the three articles did not mention the sex of patients, the participants were almost 242 men and 239 women.

### Prevalence of AIEC Isolate

Thirteen studies reported the prevalence of AIEC isolates, of these the pooled prevalence of AIEC was 28% (95% CI: 18–39%) ranging from 4 to 55% among IBD patients, and among CD patients it was 29% (95% CI: 20–40%) ranging from 4 to 55%. From six studies that investigated the prevalence of AIEC isolates among UC patients, the pooled prevalence was 13% (95% CI: 1–30%) ranging from 3 to 40%. Moreover, the pooled prevalence of AIEC was 9% (95% CI: 3–19%) ranging from 0 to 47% among control subjects (Supplementary Figures 1–4).

### Association of Colonization by AIEC With IBD

**Figure 2:**
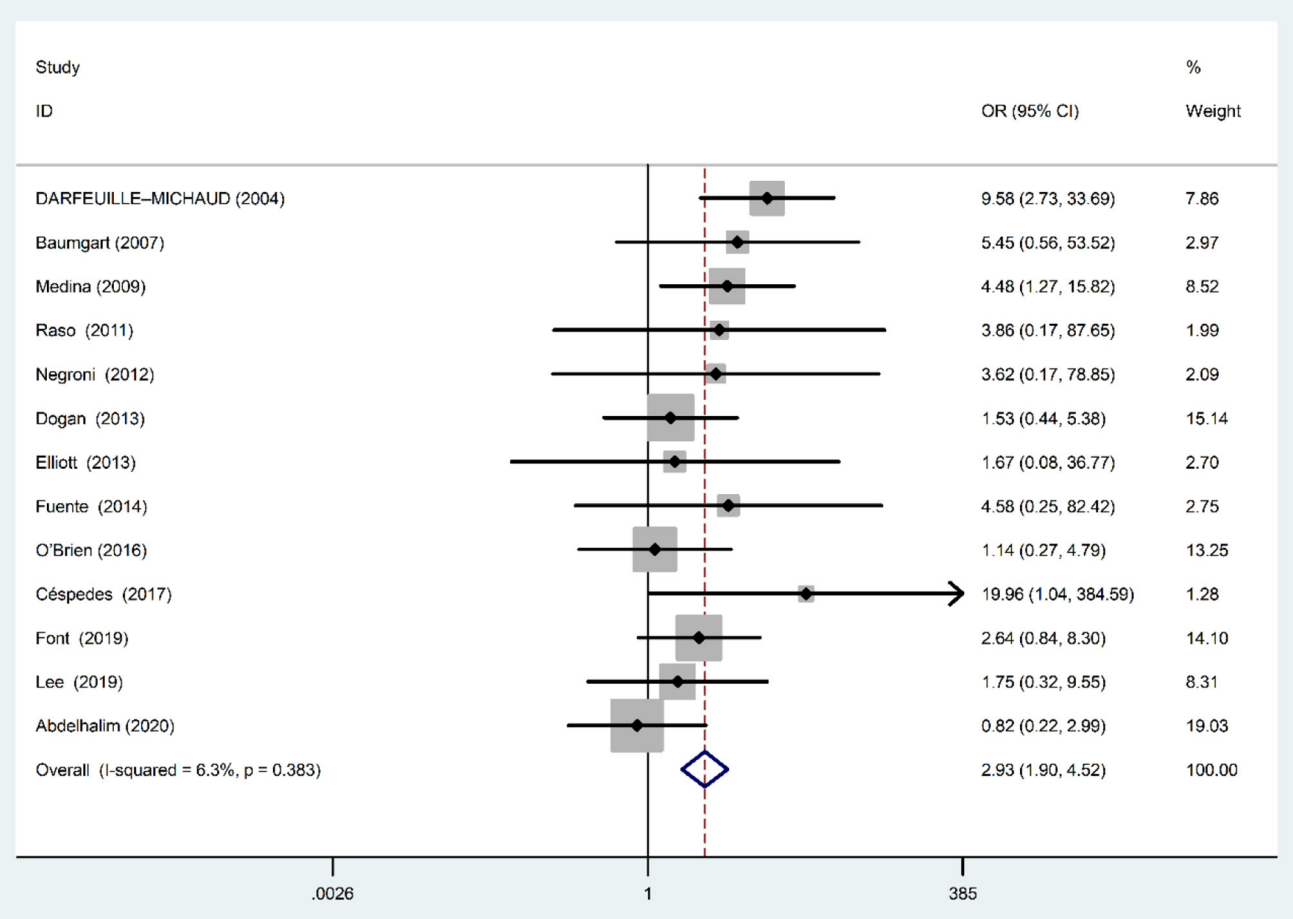
Forest plot of the association between AIEC rate and risk of IBD.

Fixed-effects meta analysis showed a significant positive association between AIEC and IBD disease (OR: 2.93; 95% CI: 1.90–4.52; *P* < 0.001) (Figure 2), indicating that the prevalence of AIEC is higher in IBD patients compared with controls. We found no evidence of heterogeneity among studies (χ^2^ = 12.81, *P* = 0.38; *I*^2^ = 6.3%).

**Figure 3:**
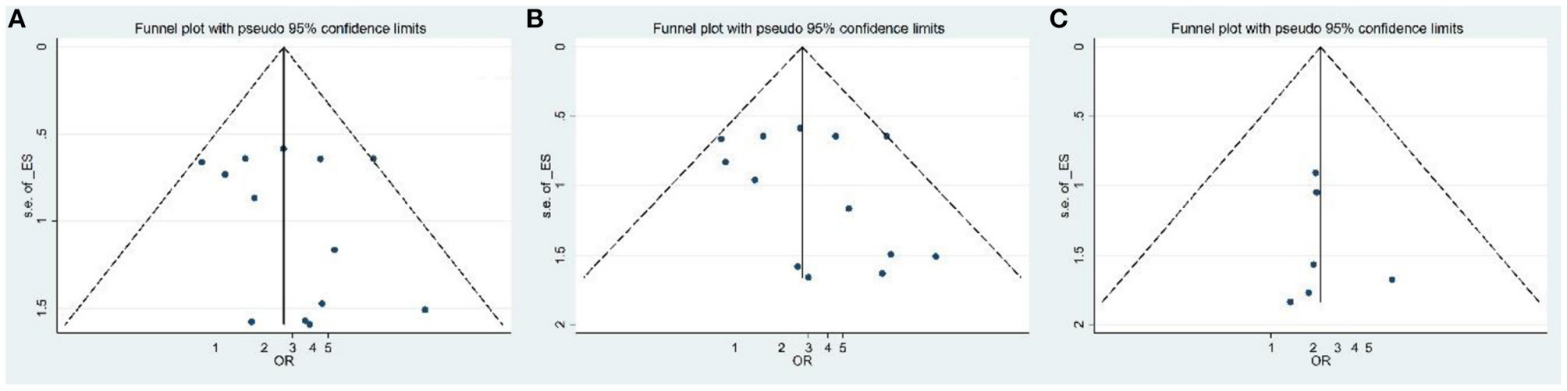
Funnel plot for evaluation of publication bias [**(A)** IBD; **(B)** CD; **(C)** UC patients].

The funnel plot for publication bias did not show any evidence of asymmetry (Figure 3A). In addition, Begg's and Egger's tests were used to quantitatively evaluate the potential publication bias. According to the results of Begg's (*Z* = 0.31, *P* = 0.76) and Egger's tests (*t* = 0.77, *P* = 0.45), there was no significant publication bias.

### Sensitivity Analysis and Meta Regression

Meta regression analysis indicated that the relationships between the association colonization by AIEC with IBD does not confound by age (OR: 1.02; 95% CI: 0.72–1.42; *P* = 0.84) (Supplementary Figure 5A). Additionally, the results of sensitivity analysis showed that none of the studies affects influentially the association of AIEC with effects of IBD (Supplementary Figure 6A). In this regard, each study was excluded and then the result was examined again. Then, no significant change in estimated pooled OR was obtained.

### Association of Colonization by AIEC With CD

**Figure 4:**
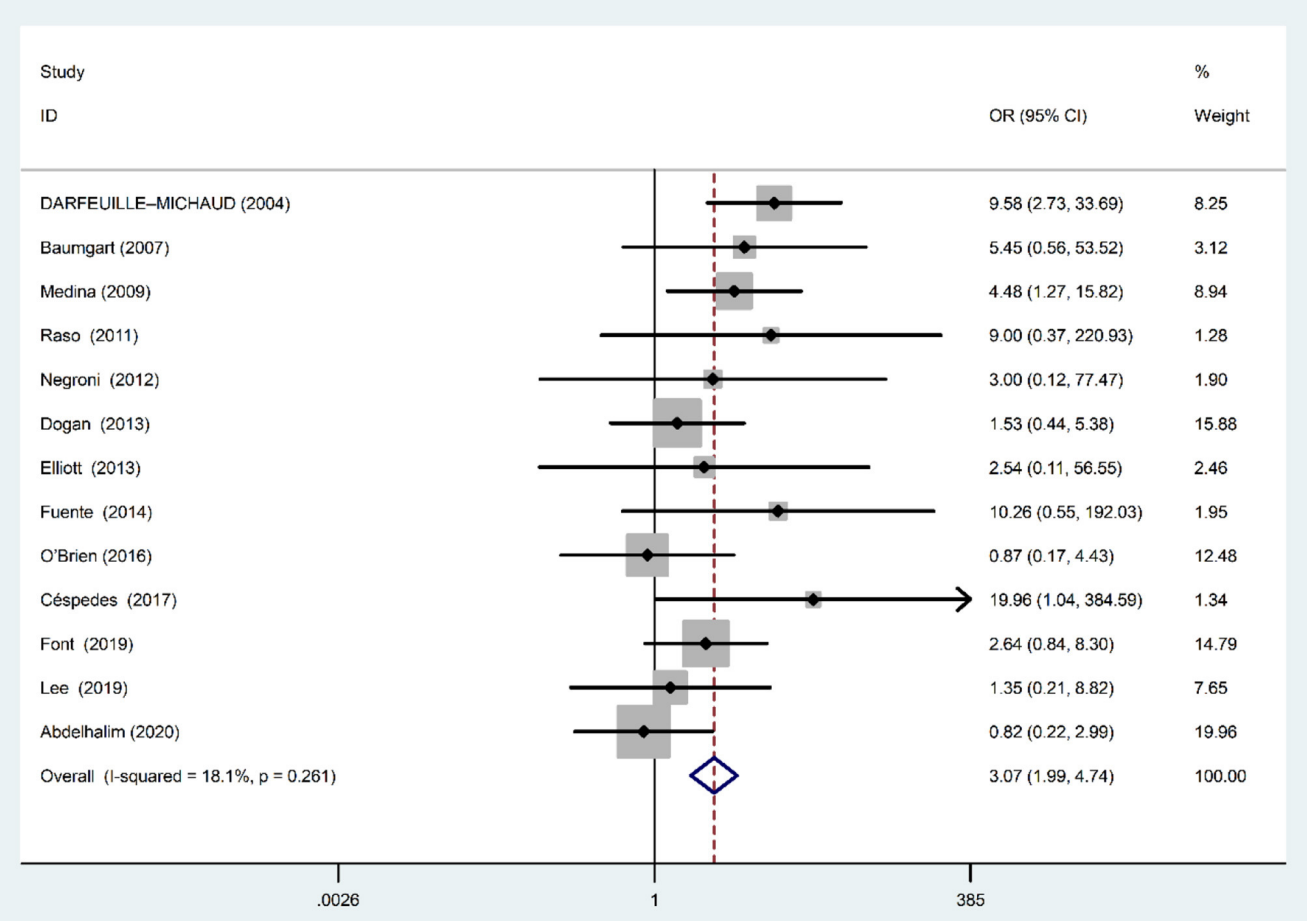
Forest plot of the association between AIEC rate and risk of CD.

Fixed-effects meta analysis showed a significant direct association between AIEC and IBD disease (OR: 3.07; 95% CI: 1.99–4.74; *P* < 0.001), indicating that the AIEC prevalence is high in patients with CD compared with controls (Figure 4). We found no evidence of between-study heterogeneity (χ^2^ = 14.66, *P* = 0.26; *I*^2^ = 18.1%).

The funnel plot for publication bias did not show any evidence of asymmetry (Figure 3B). According to the results of Begg's (*Z* = 0.79, *P* = 0.42) and Egger's tests (*t* = 0.96, *P* = 0.35), there was no significant publication bias.

### Sensitivity Analysis and Meta Regression

Meta regression analysis indicated that there is no significant relationship between age and occurrence of AIEC in patients with CD (OR: 1.07; 95% CI: 0.76–1.51; *P* = 0.66) (Supplementary Figure 5B). Additionally, the results of sensitivity analysis showed that none of the studies affects influentially the observed association of AIEC with UC patients (Supplementary Figure 6B). Each study was excluded and then the result was reevaluated. Accordingly, no significant change in estimated pooled OR was observed.

### Association of Colonization by AIEC Isolates With UC

**Figure 5:**
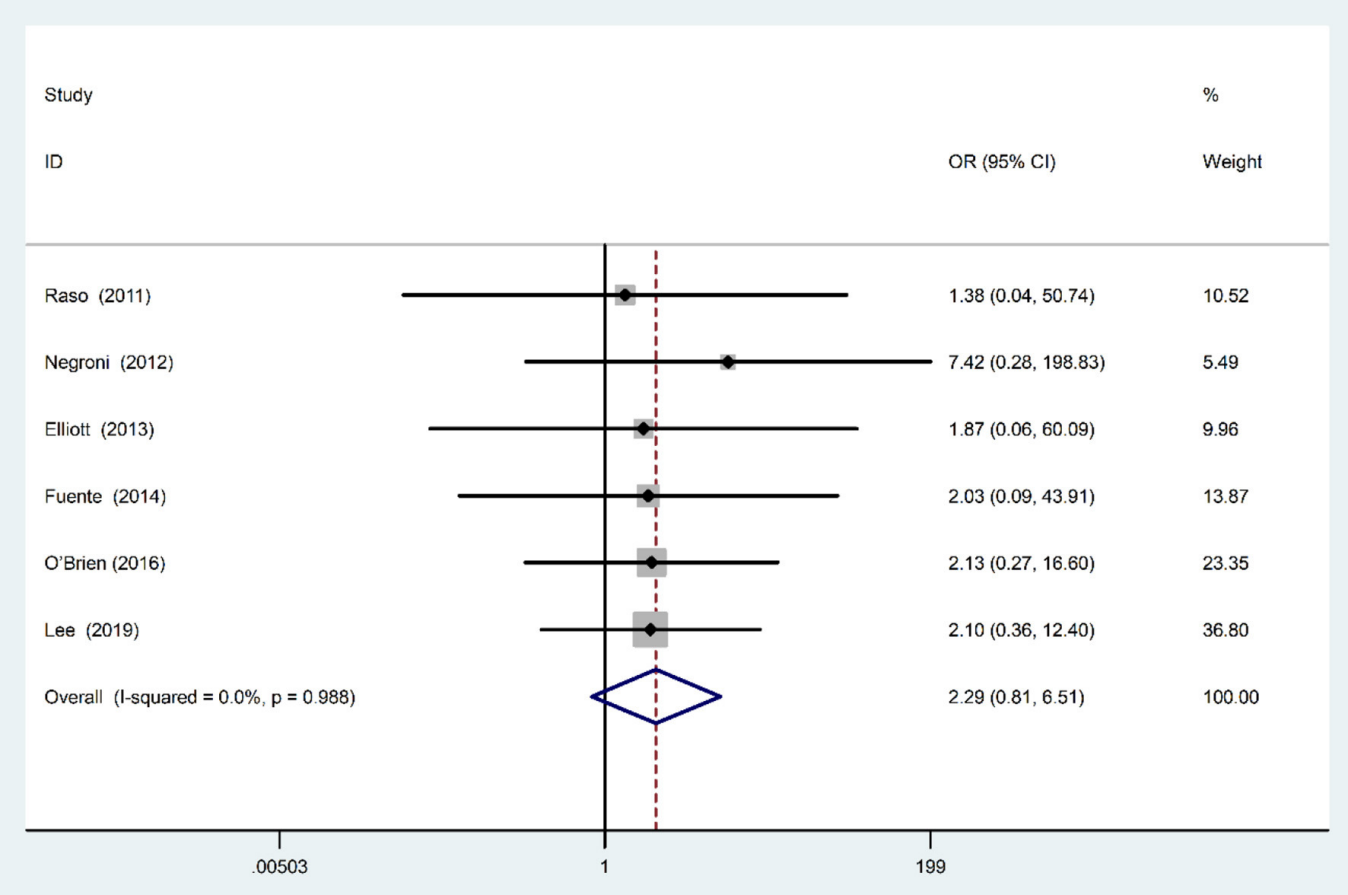
Forest plot of the association between AIEC rate and risk of UC.

Fixed-effects meta analysis showed that although based on the estimated OR the prevalence of colonization by AIEC was higher in UC patients compared with controls; however, the observed association was not statistically significant (OR: 2.29; 95% CI: 0.81–6.51; *P* = 0.11) (Figure 5). We found no evidence of between-study heterogeneity (χ^2^ = 0.6, *P* = 0.98; *I*^2^ = 0%).

The funnel plot for publication bias did not show any evidence of asymmetry (Figure 3C). According to the results of Begg's (*Z* = 0.75, *P* = 0.4) and Egger's tests (*t* = 0.34, *P* = 0.74), there was no significant publication bias.

### Sensitivity Analysis and Meta Regression

Meta regression analysis revealed a significant confounding negative effect for age in the observed association between AIEC and UC (OR: 0.83; 95% CI: 0.70–098; *P* = 0.041) (Supplementary Figure 5C). Additionally, the results of sensitivity analysis showed that none of the studies affects influentially the association between AIEC and UC (Supplementary Figure 6C). For follow up the sensitivity analysis, each study was excluded and then the result reexamined. No significant change in estimated pooled OR was obtained.

### Prevalence of AIEC Isolate Phylogenetic Groups

Eight studies reported prevalence of different phylogroup among IBD patients (Table 3). From those studies, the most prevalent phylogroup was B_2_ (53%, 95% CI: 36–69%) ranging from 20 to 100%, whereas the less prevalent was B_1_ (1%, 95% CI: 0–6%) ranging from 0 to 20%. There was no significant heterogeneity for phylogroup among the eight studies (χ^2^ = 11.99, *P* = 0.10; *I*^2^ = 41.63). Moreover, the funnel plot for publication bias did not show any evidence of asymmetry. According to the results of Begg's and Egger's tests, there was no significant publication bias among investigated phylogroup in IBD patients. The complete results of pooled prevalence, heterogeneity, and publication bias tests of different phylogenetic groups are shown in Supplementary Table 2.

**Table 3:** Phylogroups distribution among AIEC isolates.

**Authors**	**Type of disease (No. of AIEC)**	**Phylogroup**			
		**A**	**B_**1**_**	**B_**2**_**	**D**
Baumgart et al.	IBD (10)	3	2	2	3
	CD (10)	3	2	2	3
	Control (1)	0	0	0	1
Medina et al.	IBD (11)	1	0	7	3
	CD (11)	1	0	7	3
	Control (6)	2	1	3	0
Raso et al.	IBD (4)	0	0	3	1
	CD (4)	0	0	3	1
	UC (0)	0	0	0	0
	Control (0)	0	0	0	0
Elliott et al.	IBD (2)	0	0	2	0
	CD (2)	0	0	2	0
	UC (0)	0	0	0	0
	Control (0)	0	0	0	0
Fuente et al.	IBD (8)	1	0	3	4
	CD (6)	1	0	2	3
	UC (2)	0	0	1	1
	Control (0)	0	0	0	0
Cespedes et al.	IBD (13)	1	0	7	5
	CD (13)	1	0	7	5
	Control (0)	0	0	0	0
Font et al.	IBD (15)	2	0	11	2
	CD (15)	2	0	11	2
	Control (6)	2	1	3	0
Lee et al.	IBD (14)	4	1	5	4
	CD (5)	1	0	3	1
	UC (9)	3	1	2	3
	Control (2)	0	1	0	1

*AIEC, adherent–invasive Escherichia coli; CD, Crohn's disease; UC, ulcerative colitis; IBD, Inflammatory bowel disease; SS, Sample size*.

Moreover, eight studies investigated prevalence of phylogroup among CD patients. From those studies, the most frequent phylogroup was B_2_ (57%, 95% CI: 40–73%) ranging from 20 to 100%, while the less frequent was B_1_ (0%, 95% CI: 0–5%) ranging from 0 to 20%. There was no significant heterogeneity for phylogroup among the eight studies. Moreover, the funnel plot for publication bias did not show any evidence of asymmetry. According to the results of Begg's and Egger's tests, there was no significant publication bias among investigated phylogroup in CD patients (Supplementary Table 2).

In addition, among four studies that investigated prevalence of different phylogroup among control group, the highest phylogroup was B_2_ (36%, 95% CI: 8–68%) ranging from 0 to 50%, whereas the lowest was D (11%, 95% CI: 0–62%) ranging from 0 to 100%. There was no significant heterogeneity against phylogroup among the four studies. Moreover, the funnel plot for publication bias did not show any evidence of asymmetry. According to the results of Begg's and Egger's tests, except phylogroup D, there was significant publication bias among investigated phylogroup in the control group (Supplementary Table 2).

## Discussion

The current study was a comprehensive systematic review, and meta analysis was conducted to investigate the association of colonization by AIEC with IBD, IBD UC, and DC types. Based on previous studies, the mucosa-associated *E. coli* may be important in the pathogenesis of IBD, UC, and CD.

Additionally, AIEC has the ability to invade Peyer's patches and the lamina propria through M cells (44). AIEC could be adopted into macrophages, replicate, and survive within them because of the host autophagy defect. It then triggers the secretion of TNF-α through activating infected macrophages and increasing proinflammatory cytokine expression (45). Based on the studies reviewed, the incidence of IBD between colonized individuals with AIEC was different. These differences may be explained by the distribution and composition of different intestinal microbiota depending on the involvement of host and/or environmental factors (43).

In recent years, several studies have shown the role of intestinal microbiota in the development of IBD. It has been shown that in patients with IBD, the balance of intestinal bacteria is disturbed and the number of beneficial bacteria such as Bifidobacteria, Lactobacilli, and Firmicutes is reduced and the number of possible pathogenic bacteria such as Bacteroides and *E. coli* is increased (46). Studies have shown that an increase in Bacteroides and *E. coli* and a change in intestinal microbiota composition due to a high-fat/high-sugar diet increases the sensitivity to AIEC and intestinal inflammation in CEABAC10 transgenic mice (34, 45).

Majority of included studies in our systematic review and meta analysis showed significant association between IBD and the presence of AIEC; however, there were few studies with insignificant results. The non-significant results in these studies may be attributed to the low sample size and high type 2 statistical error rate. Totally, the estimated pooled OR from all included studies in our meta analysis resulted in significant relationship between AIEC rate and IBD, in which the strength of association was 2.93 and 3.07 for IBD (irrespective of disease type) and IBD CD type, respectively.

Based on literature review, individuals with high levels of CEACAM6 and CHI3L1 receptors, which are overexpressed in inflammation, promote AIEC adhesion and invasion to IECs located in the ileum *via* the type-1 pili's FimH adhesion (44, 47) or colonic IECs *via* the chitinase ChiA (48), and subsequently, the colonization of AIEC strains promote the secretion of IFN-γ and TNF-α by macrophages, which are likely to stimulate granuloma formation and is a common histological feature of CD (45). Overall, these findings suggest that AIEC strains in CD patients can promote their own colonization and the ensuing inflammatory amplification cycle (47).

Meta regression analysis indicated the association of AIEC with IBD and CD does not significantly confound by age.

Our results suggested that the affect by IBD in patients colonized with the AIEC is not random, it is a specific disease-related pathovar. That is because this pathovar has the ability to invade epithelial cells and attach to receptors of CEACAM with specificity for the oncofetal carbohydrate antigens that are overexpressed by mucosal glycoconjugates in the inflammation condition. AIEC is pertinent in IBD due to the contribution of genetic mutations associated with defects in handling intracellular microbes in the disease pathogenesis and to the intestinal injury. Thus, the mucosal environment of an individual susceptible to IBD could be exploited by these bacteria; otherwise, their proliferation might be an outcome of a normal flora depletion (49).

However, in patients undergoing AIEC colonization, the use of antibiotics may be effective in certain conditions. Antibiotics such as ciprofloxacin and rifaximin are safer alternatives for patients with CD concomitant AIEC because they have fewer side effects than immunosuppressive drugs (50). On the other hand, heptilmenoside derivatives have been shown to have strong antiadhesion effects and *in vivo*/*in vitro* protective effect against colitis, which means that they can be useful compounds for the treatment of patients with AIEC colonized patients (51). Moreover, according to previous report, the effect of diet on intestinal homeostasis and AIEC severity suggests that combined dietary use can be used to alter the availability of luminal nutrients, along with drug therapies, to limit AIEC growth and implantation (17).

In the present work, *E. coli* strains included A, B_1_, B_2_, and D phylogroups based on the availability of *chuA, yjaA*, and *TspE4.C2* genes (52). These were described on the basis of their multilocus enzyme electrophoresis patterns (MLEE). Subsequently, they were grouped by DNA-based multilocus sequence typing (MLST), and whole genome sequences confirmed it (53). B_2_ and D groups were the main parts of extraintestinal pathogenic strains of *E. coli*. However, A and B_1_ groups were the lowest pathogenic *E. coli* strains and are defined as non-human enteropathogenic strains (54).

Researchers have recently compared non-AIEC and AIEC strains of the same phylogroup and identified three genomic regions in all the B_2_ phylogroup AIEC strains, which are absent in AIEC strains of other phylogroups and commensal strains with any phylogenetic origin (e.g., B_2_) (12). Nevertheless, it is not known if these regions are only specifically present in B_2_-AIEC strains or are also available in other pathogenic groups with the same phylogenetic origin, like B_2_ ExPEC strains (12). Our results implied that most frequent AIEC phylogroup in the IBD, CD, and control groups was B_2_ and the least frequent phylogroup in the IBD and CD was B_1_, which was D in the control group. Therefore, it could be concluded that AIEC strains belonging to phylogenetic groups B_2_ might have the ability to colonize and survive in epithelial cells and macrophages in patients, particularly those with *chuA* gene.

## Conclusions

In summary, this meta analysis revealed that colonization by AIEC is more prevalent in IBD. Our meta-analysis results indicated that there is a significant association between colonization by AIEC with IBD totally and IBD CD type. In addition, the most prevalent AIEC phylogroup among the IBD patients was B_2_ and the least prevalent one was B_1_. Meanwhile, the most frequent AIEC phylogroup among the control group was B_2_ and the least frequent one was D. Our results suggested that the affect by IBD in patients colonized with the AIEC pathovar is not random, it is in fact a specific disease-related pathovar. Based on our findings, AIEC is neither commensal nor a real pathogenic strain, but it is a pathobiont that expands rapidly in the host and can apply special pathogenic influences. Although, our study showed a significant association between colonization by AIEC and IBD, these findings are based on case–control studies therefor the cause-and-effect relationship as well as directional dependency cannot be inferred. Longitudinal prospective studies will provide more reliable evidence about the directional association. However, these findings provide evidence on the importance of this strain in the treatment of IBD patients.

## Data Availability Statement

The original contributions presented in the study are included in the article/Supplementary Material, further inquiries can be directed to the corresponding author.

## Ethics Statement

This study used publicly available data. The studies involving human participants were reviewed and approved at the time of original publications (please see references and data availability statement). Written informed consent was not necessary to obtain for this study.

## Author Contributions

HF and AF conceived the study. AF, RK, MH, and PA wrote the study protocol and data analysis plan. RK and MH did the systematic review, requested individual participant data, and did the study quality assessments. AF did the statistical analysis. AF, HF, MH, RK, and PA interpreted the data. MH, RK, HF, and AF wrote the first draft of the manuscript. All authors reviewed and approved the final manuscript and had full access to all the data in the study and final responsibility for the decision to submit for publication.

## Funding

This study was supervised by AF and supported in part by a grant from Isfahan University of Medical Sciences [Grant no. 199279, Ethics Code: IR.MUI.RESEARCH.REC.1399.287].

## Conflict of Interest

The authors declare that the research was conducted in the absence of any commercial or financial relationships that could be construed as a potential conflict of interest.

## Publisher's Note

All claims expressed in this article are solely those of the authors and do not necessarily represent those of their affiliated organizations, or those of the publisher, the editors and the reviewers. Any product that may be evaluated in this article, or claim that may be made by its manufacturer, is not guaranteed or endorsed by the publisher.
